# Robust multi-task feature selection with counterfactual explanation for schizophrenia identification using functional brain networks

**DOI:** 10.3389/fnins.2025.1609547

**Published:** 2025-07-21

**Authors:** Xinyan Yuan, Shaolong Wei, Ying Sun, Lingling Gu, Yanyan He, Tiantian Chen, Hongcheng Yao, Haonan Rao

**Affiliations:** ^1^School of Electronics and Information, Jiangsu Vocational College of Business, Nantong, China; ^2^School of Artificial Intelligence and Computer Science, Nantong University, Nantong, China; ^3^School of Information Science and Technology, Nantong University, Nantong, China

**Keywords:** schizophrenia, functional connectivity, rs-fMRI, feature selection, counterfactual explanation

## Abstract

**Introduction:**

Functional brain networks measured by resting-state functional magnetic resonance imaging (rs-fMRI) have become a promising tool for understanding the neural mechanisms underlying schizophrenia (SZ). However, the high dimensionality of these networks and small sample sizes pose significant challenges for effective classification and model generalization.

**Methods:**

We propose a robust multi-task feature selection method combined with counterfactual explanations to improve the accuracy and interpretability of SZ identification. rs-fMRI data are preprocessed to construct a functional connectivity matrix, and features are extracted by sorting the upper triangular elements. A multi-task feature selection framework based on the Gray Wolf Optimizer (GWO) is developed to identify abnormal functional connectivity (FC) features in SZ patients. A counterfactual explanation model is applied to reduce perturbations in abnormal FC features, returning the model prediction to normal and enhancing clinical interpretability.

**Results:**

Our method was tested on five real-world SZ datasets. The results demonstrate that the proposed method significantly outperforms existing methods in terms of classification accuracy while offering new insights into the analysis of SZ through improved feature selection and explanation.

**Discussion:**

The integration of multi-task feature selection and counterfactual explanation improves both the accuracy and interpretability of SZ identification. This approach provides valuable clinical insights by revealing the key functional connectivity features associated with SZ, which could assist in the development of more effective diagnostic tools.

## 1 Introduction

Schizophrenia (SZ) is a chronic, often disabling mental disorder that affects one percent of the world's population (Insel, 2010; McCutcheon et al., 2020). Patients' clinical symptoms manifest in perception, thinking, and emotion, such as hallucinations, delusions, incoordinated excitement, and anxiety (Song et al., 2023; Rantala et al., 2022). Although the pathogenesis of SZ is still unclear, it is increasingly recognized that analyzing the brain network of SZ can help improve differential diagnosis and understand the pathological mechanism (Zhang et al., 2021). Recent studies have shown that functional brain networks measured by resting-state functional magnetic resonance imaging (rs-fMRI) have become a promising tool to reveal the underlying neural mechanisms of SZ (Zhu et al., 2024; Chyzhyk et al., 2015). SZ causes widespread changes in functional brain networks, including changes in global brain topology, abnormal connectivity in local regions, and the formation of specific abnormal subgraphs (Huang et al., 2025).

However, although functional brain networks provide rich pathological information, these data often have high-dimensional characteristics, making analysis and modeling face great challenges (Mhiri and Rekik, 2020). Therefore, feature selection (FS) becomes an indispensable step, which can remove irrelevant or redundant features and retain only the most diagnostically valuable information (Naheed et al., 2020). In addition, functional brain network data usually face the problem of small samples. Due to the high cost of data acquisition, the long experimental cycle, and the difficulty in recruiting subjects, the number of samples is often much lower than the feature dimension, making model training susceptible to overfitting, thereby reducing generalization ability (Turner et al., 2018; Ding et al., 2024). In this context, robust and effective FS is vital. In fact, FS plays a key role in identifying meaningful biomarkers, such as functional connectivity between brain regions, which can characterize abnormalities in brain function associated with brain diseases such as SZ, thus providing insight into understanding the neural basis of brain diseases, as well as diagnosis and prediction (Xing et al., 2022).

For functional brain network data, the traditional FS method often exhibits poor robustness across datasets, primarily due to the high dimensionality of the feature space and the scarcity of training samples, and it is difficult to identify connection features with consistency and biological interpretability (Wang et al., 2015; Lv et al., 2015; Hu et al., 2021). At present, most existing FS methods have combined advanced technologies such as machine learning or deep learning to improve performance, such as using graph neural networks to model FC structures, or improving feature selection efficiency through embedded FS strategies, but these methods still have obvious limitations. On the one hand, many models still lack consistent evaluation across data sets, making it difficult to identify robust disease-related connection features (Chan et al., 2024); on the other hand, most existing methods are black-box in form and lack interpretability, especially in clinical applications. It is difficult to provide actionable explanations or intervention recommendations (Verma et al., 2023). In addition, although some studies have introduced multimodal or high-order connection features in SZ diagnosis, it is still difficult to achieve a good balance between model generalization and explanatory power (Sunil et al., 2024).

**Figure 1:**
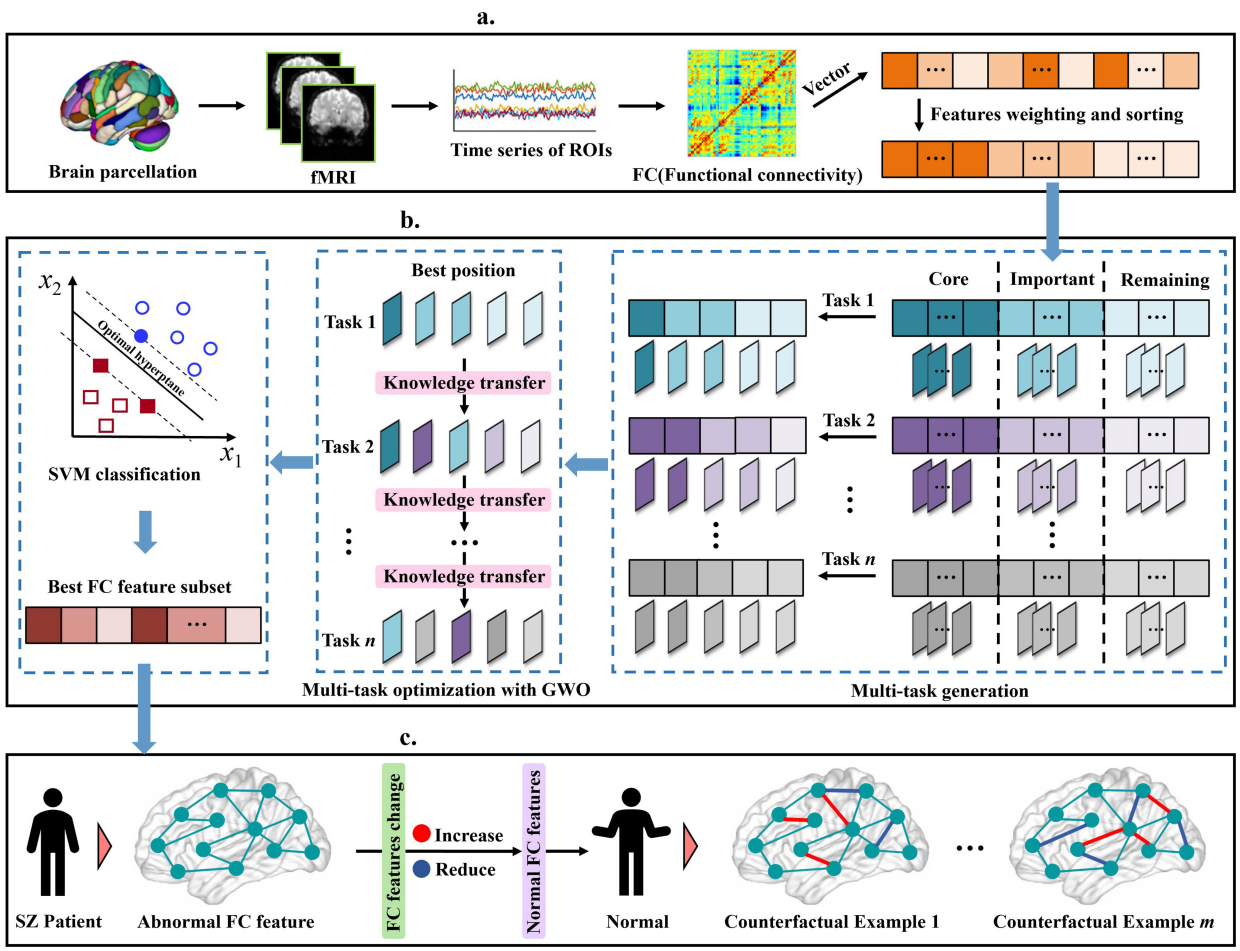
Illustration of our proposed schizophrenia analysis method, including **(a)** data pre-processing, **(b)** Robust Multi-Task Feature Selection, **(c)** Diversity counterfactual explanation.

To address the above challenges and fill this gap, we proposed a novel and robust multi-task feature selection method for SZ diagnosis, and explained the changes in brain functional connectivity (FC) caused by the disease through a counterfactual explanation model. The schematic diagram of our proposed method is shown in Figure 1. Specifically, we first preprocessed the rs-fMRI data, constructed the FC matrix, and then extracted the upper triangular elements as feature vectors and sorted them. Subsequently, we developed a robust multi-task feature selection framework based on the Gray Wolf Optimizer (GWO), and selected the abnormal FC features of SZ patients by adopting feature stratification and weight-based task generation. Finally, we used the counterfactual explanation model to generate a set of counterfactual examples for SZ patients, that is, by fine-tuning the abnormal FC features of SZ patients to make their state close to normal, thus providing theoretical guidance for the analysis and diagnosis of SZ. We verified the effectiveness of our method on five real SZ datasets, and the results showed that our method not only improved the interpretability of the model, but also provided a new perspective for the analysis of SZ. The main contributions of this paper are as follows:

- We propose a Robust Multi-Task Feature Selection with Counterfactual Explanation for Schizophrenia Identification to assist SZ analysis and diagnosis.
- We construct a multi-task feature selection framework based on GWO and combine it with the counterfactual explanation model to fine-tune the abnormal FC features of SZ patients to make their status closer to that of healthy individuals, thereby improving the accuracy of SZ classification and the interpretability of the model.
- We evaluate the performance of the proposed method using five real SZ datasets. The results show that the proposed method outperforms existing methods.

## 2 Related work

### 2.1 Gray wolf optimizer

Gray Wolf Optimizer (GWO) (Mirjalili et al., 2014) is an intelligent optimization algorithm that simulates the hunting behavior of gray wolf groups. In the context of multitasking, GWO provides efficient global search capabilities and information-sharing mechanisms between individuals, which can improve optimization performance in a multi-task environment.

Gray wolf packs are generally divided into four levels: (i) α is the leader of the wolf pack, representing the current optimal solution, (ii) β is the second-level wolf, assisting α in decision-making, representing the second-best solution, (iii) δ is the third-level wolf, assisting β, representing the third-best solution, and (iv) θ is an ordinary wolf that obeys other high-level wolves and represents the remaining candidate solutions. When searching for prey, gray wolves will gradually approach the prey and surround it:


(1) $$\begin{array}{l} D=|C\cdot X_{p}-X| \end{array}$$

(2) $$\begin{array}{l} X\left(t+1\right)=X_{p}-A\cdot D \end{array}$$

where *X*_*p*_ is the location of the prey or the current optimal solution, *X* is the location of the individual wolf, *t* is the number of iterations, and *A* and *C* are coefficient vectors, which are calculated as follows:


(3) $$\begin{array}{ll} A=2d\cdot r_{1}-d, & C=2r_{2} \end{array}$$

where *d* is the convergence factor that decreases linearly with the number of iterations, from 2 to 0, and *r*_1_ and *r*_2_ are random numbers between [0, 1]. GWO uses three optimal solutions (α, β, δ) to jointly guide the search:


(4) $$\begin{array}{l} X\left(t+1\right)=\frac{1}{3}\underset{i=\alpha ,\beta ,\delta }{\sum }\left(X_{i}-A_{i}\cdot D_{i}\right) \end{array}$$

where *D*_*i*_ = |*C*_*i*_·*X*_*i*_ − *X*|, *i* ∈ {α, β, δ}. When |*A*| becomes smaller (approaches 0), the search range is reduced, and the wolf pack gradually converges to the optimal solution. When |*A*| > 1, the wolf pack stays away from the prey and performs a global search to avoid falling into the local optimum.

### 2.2 Counterfactual explanation

Counterfactual explanations are a method for making machine learning models more transparent by showing how to change attributes to obtain different results (Spreitzer et al., 2022). Cheng et al. (2020) introduced counterfactuals with a classic example: A person submitted a loan request but was rejected by the bank. If his credit score had been 700 instead of 600, his loan application would have been approved.

Counterfactual explanations are currently widely used in different fields, including medical diagnosis, decision reasoning, and artificial intelligence. Richens et al. (2020) have improved the application of machine learning in the field of medical diagnosis, especially in identifying rare diseases, by establishing a counterfactual causal diagnosis model. Prado-Romero et al. (2023) use counterfactual explanations to provide a way to understand model decisions by providing specific changes in input features to explain the model's decision-making process. In addition, counterfactual explanations also have many applications in brain networks. For example, in the study of Abrate and Bonchi (2021), they proposed an explanation method for a black-box graph classifier for brain network classification. By analyzing counterfactual graphs, brain region connection patterns associated with specific brain region diseases can be identified. Matsui et al. (2022) proposed a new generative deep neural network (DNN) called Counterfactual Activation Generator to provide counterfactual explanations for DNN-based brain activation classifiers.

Counterfactual explanation has emerged as an important branch in the field of machine learning interpretability; however, it has not yet been applied to FC analysis. In this work, we introduce a counterfactual perspective: if the abnormal FC between brain regions in SZ patients is adjusted toward the normal range, their predicted state may shift closer to that of healthy individuals. Such counterfactual reasoning is particularly valuable in the medical domain, as it can assist clinicians in evaluating the potential impact of different treatment strategies, especially in the context of brain diseases.

## 3 Materials and methods

### 3.1 Schizophrenia dataset

In this study, five public datasets are used, including the Center for Biomedical Research (COBRE) dataset (120 subjects), the Huaxi dataset (311 subjects), the Nottingham dataset (68 subjects), the Taiwan dataset (131 subjects) and the Xiangya dataset (143 subjects). All subjects met the following conditions: (i) no other Diagnostic and Statistical Manual of Mental Disorders (DSMIV) disease exists, (ii) no history of drug abuse, (iii) no clinically significant head trauma. The specific information of the subjects is presented in Table 1.

**Table 1:** Characteristics of subjects in the five datasets in this study.

**Datasets**	**Class**	**Gender (M/F)**	***P*-value of gender**	**Age (years)**	***P*-value of age**
COBRE	NC	46/21	0.1927	34.82+11.28	0.3987
	SZ	42/11		36.75+13.68	
Huaxi	NC	79/71	0.6748	27.80+12.50	1.000
	SZ	80/81		27.80+12.50	
Nottingham	NC	26/10	0.2277	33.38+8.98	0.9855
	SZ	27/5		33.34+9.05	
Taiwan	NC	25/37	0.2329	29.87+8.62	0.2847
	SZ	35/34		31.59+9.60	
Xiangya	NC	35/25	0.9333	27.17+6.64	0.1025
	SZ	49/34		23.37+7.83	

NC, normal control; SZ, schizophrenia.

### 3.2 Data pre-processing

The rs-fMRI data of the five datasets are collected by different types of scanners, including COBRE and Xiangya by 3-T Siemens Tim-Trio scanner with an eight or 12-channel head coil, Huaxi by 3-T General Electric MRI scanner, and Nottingham by 3-T Philips Achieva MRI scanner. The rs-fMRI data are preprocessed using the program standard procedures of SPM 8 and the Data Processing Assistant for Resting-State fMRI (DPARSF). The following steps are performed: (i) removing the first 10 volumes, (ii) slice timing correction, (iii) head motion correction, (iv) regress out the nuisance covariates, (v) normalized to standardized space, (vi) voxel-wise bandpass filtering, (vii) normalization of anatomical images to MNI template space, and (viii) smoothing with a 4 mm Full Width at Half Maximum (FWHM) Gaussian kernel. After processing, we defined the nodes of the brain network according to the Automatic Anatomical Labeling (AAL) template, and calculated the pairwise similarities between the noded1s of the time series as the connecting edges of the brain network.

Next, let $A_{i}^{F}\in {\mathbb{R}}^{N\times N}$ be the connectivity matrix of the functional brain network, *N* be the number of regions of the brain network, *i* = 1, 2, ..., *p*, and *p* be the number of subjects. We take the upper triangular elements of the matrix as features and represent them as vectors $S_{i}=\left(s_{i}^{1},...,s_{i}^{j},...,s_{i}^{q}\right)\in {\mathbb{R}}^{1\times q}$, $q=\frac{N\left(N-1\right)}{2}$, $s_{i}^{j}$ represents the *j*-th feature of the *i*-th subject, and *Y*_*i*_ ∈ ℝ is the label of the *i*-th subject. It is worth noting that in this paper, we divided the brain network into 90 regions of interest (ROI), that is, *N* = 90, so each subject contains a vector of dimension 1 × 4,005, which reflects the functional connectivity strength pattern between the 90 brain regions of the subject.

### 3.3 Robust multi-task feature selection

#### 3.3.1 Multi-task generation

To identify the most critical FC features for brain disease diagnosis, we use the infinite feature selection (IFS) (Roffo et al., 2020) method to calculate the importance of each feature and rank the features accordingly. Specifically, the weight of each feature is calculated based on the linear weighting of the following three aspects (i.e., Fisher criterion *h*_*j*_, mutual information *m*_*j*_, and standard deviation σ_*j*_). The first is the Fisher criterion:


(5) $$\begin{array}{l} h_{j}=\frac{|\mu_{j,1}-\mu_{j,2}{|}^{2}}{\sigma_{j,1}^{2}+\sigma_{j,2}^{2}} \end{array}$$

where μ_*j, g*_ and σ_*j, g*_ represent the mean and standard deviation of the *j*-th feature in the *g*-th class, respectively. In our experiments, both are binary classifications, so *g* ∈ {0, 1}.

The second is the normalized mutual information *m*_*j*_ between feature *s*^*j*^ and class label *Y*:


(6) $$\begin{array}{l} m_{j}=\underset{y\in Y}{\sum }\underset{z\in s^{j}}{\sum }u\left(z,y\right)log\left(\frac{u\left(z,y\right)}{u\left(z\right)u\left(y\right)}\right) \end{array}$$

where *Y* is the set of class labels and *u*(·) represents the joint distribution probability.

The third is the standard deviation σ_*j*_, which reflects the dispersion of feature *s*^*j*^ in the sample.

The final weight of each feature *s*_*i*_ is calculated as follows:


(7) $$\begin{array}{l} s_{i}=\alpha_{1}\cdot h_{j}+\alpha_{2}\cdot m_{j}+\alpha_{3}\cdot \sigma_{j}. \end{array}$$

where α_1_ + α_2_ + α_3_ = 1, this weighting approach allows us to flexibly adjust the contribution of each indicator in the selection of features, thus selecting the most informative features for the diagnosis of schizophrenia (SZ).

Based on the preliminary evaluation of FC feature importance based on the above three factors, we further constructed a feature weight curve and optimized the FS process by introducing a knee point detection algorithm, following the knee point detection method proposed by Chen et al. (2021). This approach provides an automated criterion for determining the optimal feature subset size. Specifically, after obtaining the weight of each feature, we first construct a straight line connecting the starting point and the end point of the weight curve, and then calculate the vertical distance from each point on the curve to the straight line. The knee point (*x*_*knee*_, *y*_*knee*_) is the point that maximizes the distance:


(8) $$\begin{array}{l} \left(x_{knee},y_{knee}\right)=\arg\max_{j}\left(\frac{\left|y_{j}-\left(ax_{j}+b\right)\right|}{\sqrt{a^{2}+1}}\right) \end{array}$$

where *a* and *b* are the slope and intercept of the straight line determined by the starting point and the end point, (*x*_*j*_, *y*_*j*_) is the coordinate of the *j*-th feature point on the curve, *j* = 2, 3, ..., *q* − 1. The identified knee points divide the feature weight curve into multiple intervals, and the features in each interval are given different priorities according to their weights.

Based on the location of the knee points, as shown in Figure 1b, we divide the features into three categories:

(i) Core features: located before the first knee point. These features are usually highly correlated with the predicted target variable and have low redundancy, and contribute the most to the model's predictive ability.
(ii) Important features: located between the two knee points. Although these features are not as important as the core features, may still contain useful information for specific scenarios. When combined with other features, they can enhance overall model performance, especially in complex cases where feature interactions are significant.
(iii) Remaining features: located after the second knee point. These features contribute less to the prediction task, contain redundant information, or have low correlation with the target variable.

After the above steps, we further use this category information to guide the task generation process. To ensure that the feature extraction process not only reflects its relative importance but also maintains appropriate diversity, we adopt a probabilistic extraction method based on feature weights. Specifically, we determine the initial selection probability of each feature based on the feature weight.


(9) $$\begin{array}{l} P_{j}=\frac{\omega_{j}}{\sum_{j=1}^{q}\omega_{j}} \end{array}$$

where ω_*j*_ is the weight of the *j*-th feature. The larger ω_*j*_ is, the higher its initial extraction probability is, and thus it is given priority in FS. To ensure that all features have a certain chance of being selected and to avoid the extraction probability of low-weight features becoming too small, we adjust the initial probability:


(10) $$\begin{array}{l} {P^{\prime }}_{j}=\frac{P_{j}}{\max\left(P_{j}\right)} \end{array}$$

The above formula ensures that the maximum extraction probability of a feature is 1, and the extraction probabilities of all other features are adjusted proportionally, avoiding excessive neglect of low-weight features while still maintaining the priority of high-weight features during extraction.

During the task generation process, a random number λ between 0 and 1 is first randomly generated, which is used to determine which features will be selected for the current task. For each feature *s*^*j*^, if $\lambda \leq {P^{\prime }}_{j}$, the feature will be selected for the current task. As shown in Figure 1b, after *n* rounds of independent extraction, *n* different task sets are generated, each of which contains a set of selected feature subsets. This mechanism ensures that high-weight features are selected first and fully retain the potential contribution of low-weight features, thereby effectively improving the diversity and flexibility of the task generation process.

#### 3.3.2 Multi-task optimization with GWO

In multi-task optimization, we propose to combine the knowledge transfer mechanism with the GWO-based multi-task optimization method to enhance information sharing between different tasks, thereby improving the efficiency and effect of overall optimization. Specifically, we directly integrate the knowledge transfer mechanism in the initialization phase of GWO to make full use of the optimization experience of existing tasks.

To achieve effective knowledge transfer, in the multi-task optimization process, we first need to quantify the importance of each feature in the previous task. In other words, we need to calculate the cumulative number of times *Q*_*KT*_ that feature *s*^*j*^ is selected in all previous tasks:


(11) $$\begin{array}{l} Q_{KT}\left(s^{j}\right)=\sum_{t=1}^{n}Q_{KT}^{t}\left(s^{j}\right) \end{array}$$

where *n* represents the total number of tasks, $Q_{KT}^{t}\left(s^{j}\right)$ represents whether the feature is selected in the *t*-th task (if selected, it is 1, otherwise it is 0). Then, calculate the probability *P*(*s*^*j*^) of feature *s*^*j*^ being selected in the initial population of the new task:


(12) $$\begin{array}{l} P\left(s^{j}\right)=\frac{Q_{KT}\left(s^{j}\right)}{\sum_{j=1}^{q}Q_{KT}\left(s^{j}\right)} \end{array}$$

**Figure 2:**
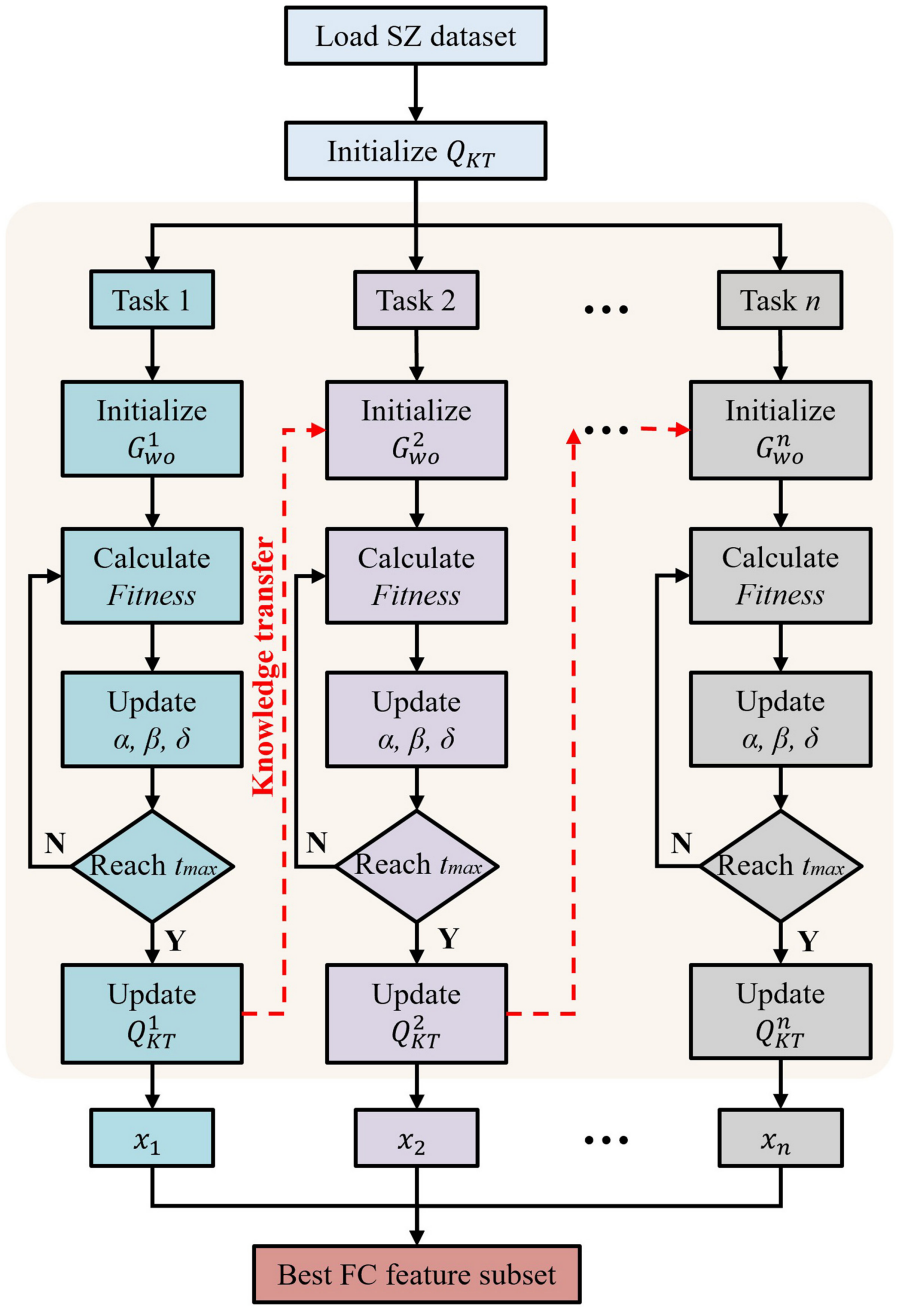
Flowchart of proposed multi-task optimization with GWO.

The above formula converts the historical performance of the feature into a probability value, which will be directly applied to initialize the wolf pack:


(13) $$\begin{array}{l} G_{wo}=\{\begin{array}{cc} 1, & \lambda \leq P\left(s^{j}\right)\\ 0, & \lambda >P\left(s^{j}\right) \end{array} \end{array}$$

where the random number λ ∈ [0, 1], the feature *s*^*j*^ is selected only when it is less than or equal to *P*(*s*^*j*^). For ease of understanding, we show the specific process of the proposed multi-task optimization method in Figure 2. First, the global environment is set. Subsequently, the algorithm enters a loop and processes *n* tasks in turn. For each task, the wolf pack is initialized independently, using the global knowledge of the previously processed tasks to provide information for the initial state of the search for the new task. The position of the wolf is iteratively updated to optimize the FS problem. After optimization, the best solution is used to update the global knowledge base. This cycle is repeated for each task, ensuring the continuous flow of information and the improvement of the solution. Finally, *n* feature subsets (*x*_1_, *x*_2_, ..., *x*_*n*_) are obtained from the *n* tasks.

In addition, to minimize the number of selected features while maintaining a high classification accuracy, we designed a fitness function in multi-task optimization and introduced a penalty term to constrain the number of features:


(14) $$\begin{array}{l} Fitness=\rho \times ACC-\left(1-\rho \right)\times \frac{q_{sf}}{q} \end{array}$$

where ρ is a weight coefficient, which ranges between [0, 1] and is used to balance the classification accuracy *ACC* and the number of selected features *q*_*sf*_.

After the above operations, we represent the selected feature matrix as *S*′ ∈ ℝ^*p* × *k*^, where *k* ≪ *q*. Based on the selected feature matrix *S*′, we can train a suitable machine learning model [i.e., *f*(·)] to predict schizophrenia. In our experiment, since the support vector machine (SVM) is strongly adaptable to small sample data sets, we used SVM as the classification model.

#### 3.3.3 Diversity counterfactual explanation

To enhance the interpretability of our method, we further introduce a counterfactual explanation model (Mothilal et al., 2020) to generate sample-level explanations. The input of this model includes a trained SVM model [i.e., *f*(·)] and the feature vector $c_{i}\in {\mathbb{R}}^{1\times k}$ of the *i*-th subject. Our goal is to generate a set of counterfactual examples $\left\{x_{i}^{1},x_{i}^{2},...,x_{i}^{L}\right\}$ for subject *i* such that its decision outcome $x_{i}^{l}\in {\mathbb{R}}^{1\times k}$ is different from the prediction of the original feature vector *c*_*i*_.

The counterfactual explanation model consists of three parts: loss function *loss*(·), distance function *dist*(·), and diversity metric *diversity*(·). Specifically, the first part pushes counterfactual $x_{i}^{l}$ toward different predictions, the second part makes counterfactual examples closer to the original input, and the third part is used to increase the diversity of counterfactual explanations. In the first part, we use a hinge loss function that helps generate counterfactuals with less variation by reducing the preference for extreme values. The hinge loss is expressed as follows:


(15) $$\begin{array}{l} loss_{hinge}=max\left(0,1-z\cdot logit(f(x)\right) \end{array}$$

where *z* is 1 when Ŷ = 1 and −1 when Ŷ = 0, and *logit*(*f*(*x*)) is the unscaled output of the SVM model. It is worth noting that in our experiments, 1 corresponds to normal subjects and 0 corresponds to patients, so in the verification of converting patients into normal subjects, Ŷ is usually set to 1. For the choice of distance function in the second part, we follow Wachter et al. (2017) proposal and divide the distance of each feature by the median absolute deviation (MAD) of the feature values in the training set:


(16) $$\begin{array}{l} dist\left(x,c\right)=\frac{1}{L}\overset{L}{\underset{\alpha =1}{\sum }}\frac{\left|x^{\alpha }-c^{\alpha }\right|}{MAD_{\alpha }} \end{array}$$

where *MAD*_α_ is the median absolute deviation of the α-th feature, *L* is the total number of counterfactual examples to generate, *x* represents the counterfactual example and *c* represents the original feature vector. For the third part, we use a determinant-based point procedure to measure the diversity of counterfactual examples, computed by the determinant value of its kernel matrix *K*:


(17) $$\begin{array}{l} diversity=det\left(K\right) \end{array}$$

where $K_{u,v}=\frac{1}{1+dist\left(x^{u},x^{v}\right)}$, *x*^*v*^ and *x*^*u*^ represent two counterfactual examples. In the experiments, to avoid uncertain determinants, we add small random perturbations on the diagonal elements to calculate the determinant.

Finally, we can obtain counterfactual examples by optimizing the following loss:


(18) $$\begin{array}{l} \;X\left(c_{i}\right)=\frac{\gamma_{1}}{L}\overset{L}{\underset{l=1}{\sum }}dist\left(x_{i}^{l},c_{i}\right)\\ \;-\gamma_{2}diversity\left(x_{i}^{1},x_{i}^{2},...,x_{i}^{L}\right)\\ +\underset{x_{i}^{1},x_{i}^{2},...,x_{i}^{L}}{\arg\min}\frac{1}{L}\overset{L}{\underset{l=1}{\sum }}loss_{hinge}\left(f\left(x_{i}^{l}\right),\hat{Y}\right) \end{array}$$

where *X*(*c*_*i*_) is the final counterfactual explanation model, γ_1_ and γ_2_ are hyperparameters for balancing the three parts of the loss function. The above formula reveals the minimum change required for the input data to achieve the idealized result. By adjusting the FC values between abnormal brain regions of SZ patients, their state may be closer to normal. This method not only provides an intuitive explanation scheme, but also provides SZ patients and doctors with the guidance needed to treat the disease.

## 4 Experiments and results

### 4.1 Experimental setting

In this work, we use a support vector machine (SVM) classifier to perform the classification task on five SZ datasets. During the experiments, we evaluate the performance of different methods based on diagnostic accuracy (ACC = $\frac{\text{TP+TN}}{\text{TP+TN+FP+FN}}$), sensitivity (SEN = $\frac{\text{TP}}{\text{TP+FN}}$) and specificity (SPE = $\frac{\text{TN}}{\text{TN+FP}}$). FP, TP, FN, and TN represent false-positive, true-positive, false-negative, and true-negative classification results. To ensure fairness, all compared FS methods use SVM classifiers. The parameters of our method are set as α_1_ = α_2_ = 0.4, α_3_ = 0.2, *t*_*max*_ = 100, ρ = 0.9, *n* = 8, *L* = 10, γ_1_ = 0.5 and γ_2_ = 1. It is worth noting that we use a five-fold cross-validation strategy in all experiments.

### 4.2 Statistical analysis of FC features

**Figure 3:**
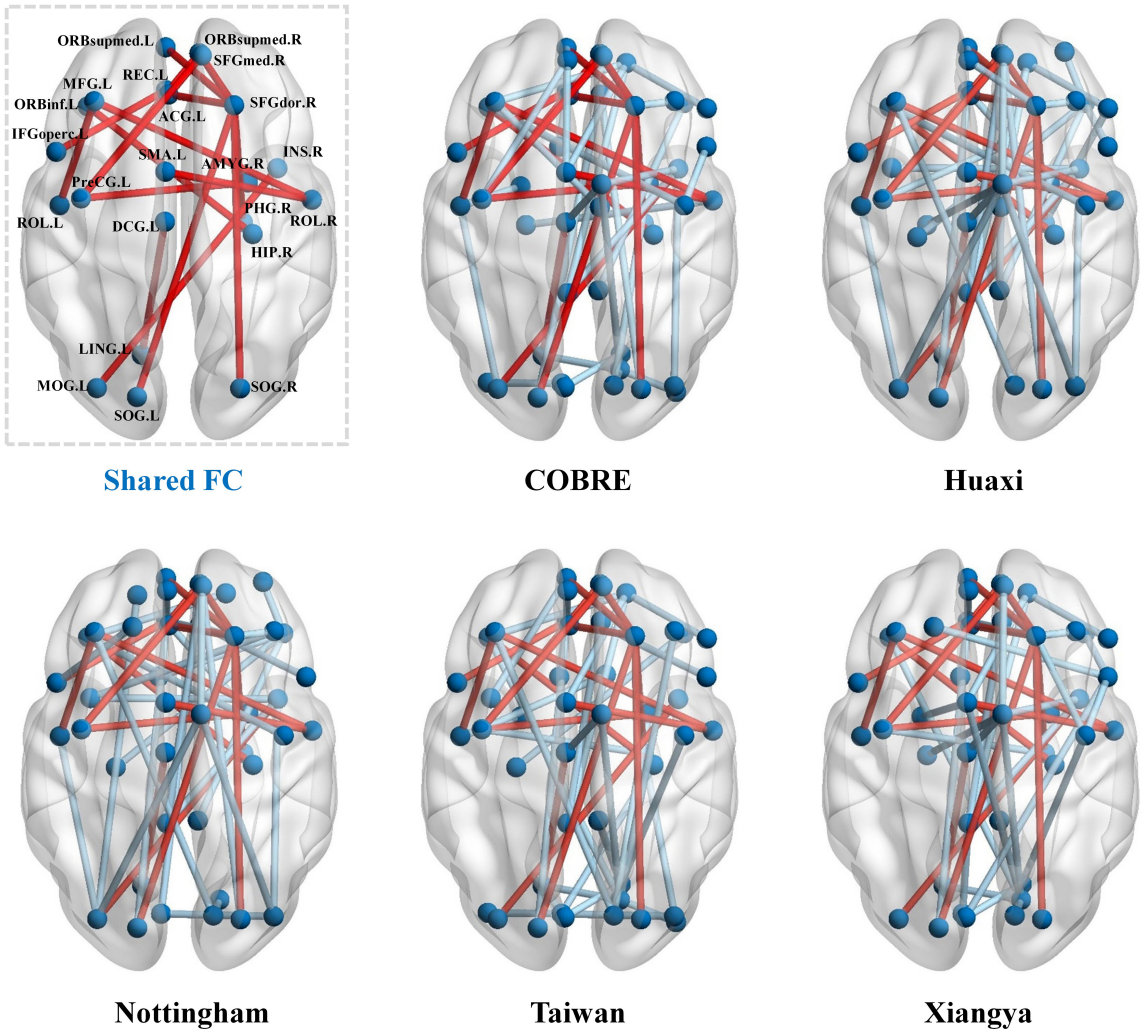
Functional connectivity (FC) retained after feature selection by our method in the five datasets. The red lines indicate the common functional connectivity among the five datasets.

In this set of experiments, we perform statistical analysis on the functional connectivity (FC) remaining after feature selection by our method to demonstrate the effectiveness of our method. For intuitiveness, we first show the FC features retained after feature selection by our method in Figure 3. As can be seen from Figure 3, there are 16 shared FCs in the five datasets, and these shared FCs are selected as features in different datasets, indicating that they are crucial in identifying SZ. In addition, these shared FCs are mainly distributed in key brain regions such as the prefrontal cortex (PFC), cingulate gyrus (CC), and hippocampus (HIP), which is consistent with the findings of existing studies on SZ in brain network abnormalities (Orellana and Slachevsky, 2013; Wei et al., 2021; Frankle et al., 2022; Haznedar et al., 2004).

**Figure 4:**
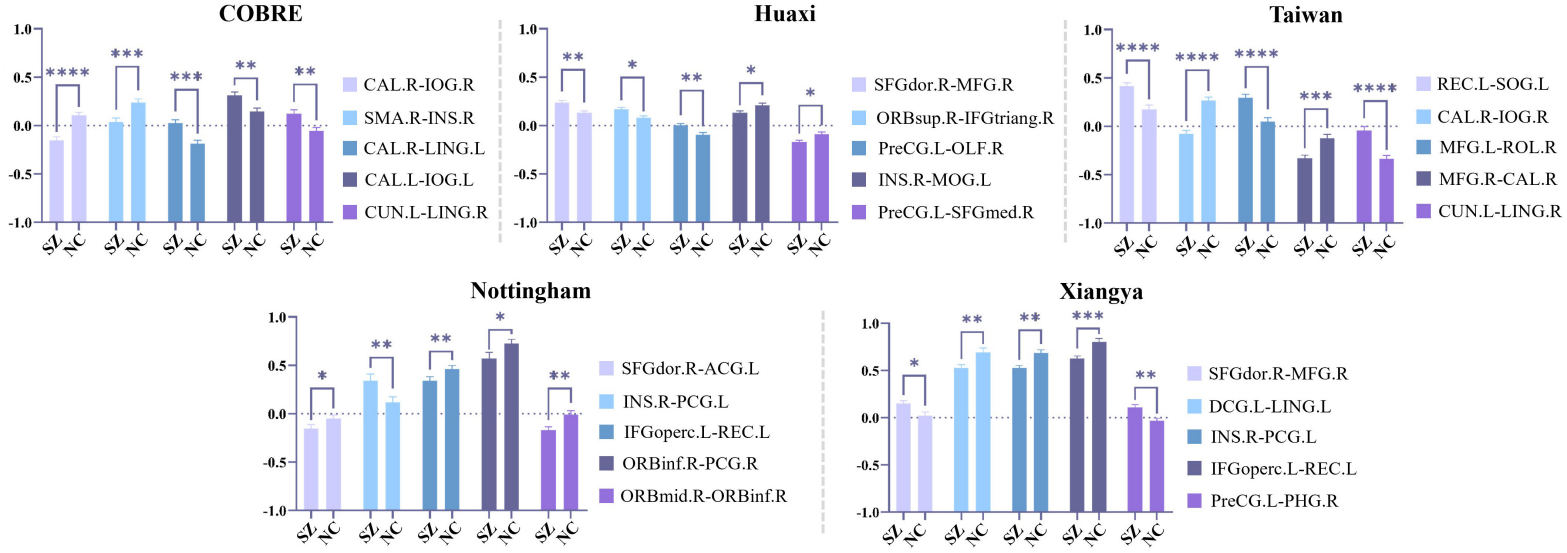
Statistical analysis is performed on the functional connections (FC) retained by feature selection using our method, and the five most statistically significant FC values between SZ and NC in the five datasets are shown here. Among them, ^*^ indicates 0.01 < *p* < 0.05, ^**^ indicates 0.001 < *p* < 0.01, ^***^ indicates 0.0001 < *p* < 0.001, and ^****^ indicates *p* < 0.0001.

We select the five most statistically significant FC values between SZ and NC based on the statistical significance of each dataset, and the results are shown in Figure 4. From Figure 4, we find that the FC values between SZ and NC show different distribution patterns in the five datasets. Specifically, in some datasets, the FC values of SZ patients are significantly higher than those of NC, while in other datasets, the FC values of SZ patients are significantly lower than those of NC. This suggests that there may be some heterogeneity in the functional connectivity patterns of SZ patients in different datasets. However, although the distribution of FC values in different datasets is different, some specific FCs show significant differences in multiple datasets, indicating that these FCs may play a key role in the neural mechanism of SZ.

Overall, the above results show that our method effectively extracts stable and biologically meaningful FC features, which helps to improve the accuracy and interpretability of SZ classification.

### 4.3 Comparison methods

We compare our proposed method with seven methods, including (i) RAW: classification without feature selection, as a baseline to illustrate the effect of applying feature selection techniques. (ii) LASSO: Lasso regression model based on L1 regularization (Cui et al., 2021). (iii) MFCSO: Multitasking Feature Selection via Competitive Swarm Optimizer (Li L. et al., 2023). (iv) MOEA\D: Multi-Objective Evolutionary Algorithm based on Decomposition (Wang et al., 2021). (v) SPEA: Strength Pareto Evolutionary Algorithm (Jiang and Yang, 2017). (vi) PSO-MET: Evolutionary Multitasking-Based Feature Selection via Particle Swarm Optimization (PSO) (Chen et al., 2020). (vii) MTPSO: Multitasking feature selection via PSO (Chen et al., 2021).

For all the above methods, the hyperparameters were set according to the values recommended in their respective original papers. Additionally, the number of iterations for all methods was set to 100, ensuring a consistent and fair comparison across all approaches.

MFCSO uses three filter methods for multi task feature selection, with each task optimized as an independent task without direct correlation between them. Therefore, the feature selection process may lack consistency. When dealing with specific datasets, especially on the schizophrenia (SZ) dataset, MFCSO may not be able to ensure consistency of selected features across different tasks, which may result in unstable performance on different datasets. Due to the lack of inter task correlation, feature selection results may be affected by randomness, making it difficult to effectively capture stable features related to schizophrenia.

Multi-objective evolutionary algorithms, such as MOEA\D and SPEA, are designed to address multiple objectives in feature selection. These algorithms provide a better balance between accuracy and feature diversity by considering multiple criteria in the optimization process. However, they are computationally intensive and can be prone to converging to local optima, especially in high-dimensional spaces. Furthermore, they often struggle with the trade-off between model complexity and accuracy, which can result in overfitting in small-sample scenarios, limiting their generalization ability.

PSO-MET and MTPSO are both particle swarm optimization-based methods that aim to improve feature selection by leveraging the concept of multitasking. While these methods are effective at identifying relevant features in some cases, they tend to be overly sensitive to initial conditions and parameter settings, leading to performance fluctuations. The lack of consistency across tasks and datasets reduces their reliability, particularly in real-world clinical settings where the data may be noisy or heterogeneous.

In comparison, our proposed method integrates robust multi-task feature selection with counterfactual explanation, offering several advantages over the methods discussed above. By using the Gray Wolf Optimizer (GWO) for feature selection, we ensure that our method not only handles high-dimensional data efficiently but also maintains stability across different datasets. The multi-task learning framework in our method allows for the sharing of knowledge across tasks, which improves generalization and reduces the risk of overfitting, particularly in small-sample situations.

### 4.4 Parameter analysis

**Figure 5:**
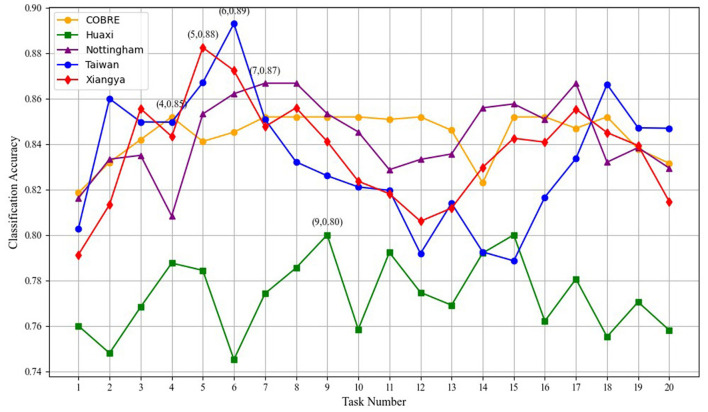
Impact of varying task numbers on model performance.

In this section, we investigate the impact of varying the number of tasks on the performance of our multi-task optimization framework, as shown in the Figure 5. We observe that increasing the number of tasks generally leads to improvements in classification accuracy, especially for datasets such as Taiwan and Xiangya. These datasets achieve their highest classification accuracy at around six–nine tasks, where the accuracy reaches 0.87 and 0.89, respectively. This indicates that knowledge sharing between tasks is particularly effective in enhancing model performance when the task number is moderate. However, beyond a certain point, specifically around 10–12 tasks, the performance begins to plateau, with only marginal improvements in classification accuracy. The graph clearly shows that the datasets, such as Xiangya and Nottingham, while still improving with increasing task numbers, experience diminishing returns as the number of tasks exceeds 10. This suggests that while task number does play a role in boosting performance, there is an optimal task count that provides the best trade-off between performance enhancement and computational cost.

A deeper analysis reveals that the knowledge sharing between tasks is highly beneficial for improving classification performance. As the number of tasks increases, the model can leverage a broader range of features, which enhances its ability to generalize. However, once the number of tasks exceeds a threshold, redundancy starts to creep into the shared knowledge. This results in the transmission of features that do not contribute significantly to the performance improvement, thereby leading to a less efficient model. The redundancy of features becomes particularly evident when the number of tasks increases beyond 10, where the performance gains start to level off, and the computational overhead grows significantly.

Thus, while task quantity is crucial for leveraging task interdependencies and improving model accuracy, an excessive number of tasks may lead to inefficiency due to the sharing of redundant or less informative features. Therefore, it is essential to strike a balance between the number of tasks and the computational cost to ensure the model remains both effective and efficient.

### 4.5 Classification performance

In this set of experiments, we compare our proposed method with seven methods and show the results in Table 2. It is not difficult to see that our method shows excellent stability and consistency on the five datasets. Specifically, in the five datasets, the ACC of our method reaches 85.19% (COBRE), 80.00% (Huaxi), 86.67% (Nottingham), 89.29% (Taiwan), and 88.24% (Xiangya), while the ACC of most methods does not exceed 85%. Secondly, our method performs outstandingly in both SEN and SPE, with SPE reaching 94.74% on the Xiangya dataset and SEN reaching 82.86% on the Huaxi dataset, indicating that our method has strong stability in the ability to distinguish between positive and negative samples. PSO-MET and MTPSO perform well in terms of SEN. For example, in the COBRE dataset, the SEN of MTPSO is 83.93%, which is higher than other methods, indicating that it has a strong ability to identify positive samples. In addition, we find that the methods based on multi-task optimization and evolutionary algorithms (i.e., PSO-MET and MTPSO) perform better overall. For example, in the Xiangya dataset, the ACC of MTPSO reaches 82.79%, which is significantly higher than other methods. This can be attributed to the fact that multi-task methods utilize shared knowledge across tasks, thereby improving the overall learning process. In general, the methods based on multi-task optimization and evolutionary algorithms have higher accuracy in SZ identification, while our method shows even better performance.

**Table 2:** Classification performance comparison with existing methods.

**Datasets**	**Metric**	**RAW**	**LASSO**	**MFCSO**	**MOEA\D**	**SPEA**	**PSO-MET**	**MTPSO**	**Our method**
COBRE	ACC (%)	63.41	75.00	68.10	73.40	69.44	78.38	81.19	**85.19**
	SEN (%)	58.33	66.67	60.00	76.47	78.57	68.75	**83.93**	80.00
	SPE (%)	73.68	79.17	76.19	69.77	63.64	85.71	79.45	**91.67**
Huaxi	ACC (%)	61.29	69.89	72.31	76.60	77.66	75.53	76.74	**80.00**
	SEN (%)	55.56	70.83	64.57	80.39	80.85	69.39	78.55	**82.86**
	SPE (%)	71.43	68.89	74.81	72.09	74.47	**82.22**	74.60	76.67
Nottingham	ACC (%)	65.00	66.12	72.22	72.34	75.53	80.95	82.71	**86.67**
	SEN (%)	66.67	68.97	66.67	74.51	76.60	80.00	82.13	**85.71**
	SPE (%)	63.64	62.50	77.78	69.77	74.47	81.82	83.08	**87.50**
Taiwan	ACC (%)	70.21	79.49	77.32	77.50	80.00	85.00	81.55	**89.29**
	SEN (%)	74.47	77.27	73.68	73.68	**88.24**	80.95	78.26	87.50
	SPE (%)	65.96	82.35	80.95	80.95	73.91	89.47	84.52	**91.67**
Xiangya	ACC (%)	66.90	69.23	79.41	76.74	70.77	72.31	82.79	**88.24**
	SEN (%)	51.35	58.82	72.22	72.00	74.29	68.57	**83.58**	80.00
	SPE (%)	67.39	69.73	87.50	83.33	66.67	76.67	81.79	**94.74**

Bold values represent the optimal values.

In addition, for the statistical significance of model performance, we select the three best-performing comparison methods (SPEA, PSO-MET, and MTPSO) in the experiment, and perform paired *t*-tests on the ACC indicators of each method on multiple datasets. The results are shown in Table 3. As can be seen from Table 3, our proposed method shows statistically significant differences with the three comparison methods on all datasets (*p* < 0.05). Specifically, the comparison with the SPEA method shows extremely significant differences on the COBRE, Nottingham, and Xiangya datasets (*p* < 0.005), and the comparison with PSO-MET has *p* values less than 0.025 on all datasets, indicating that the differences are highly statistically significant. At the same time, compared with the MTPSO method, although the *p* values in some datasets (such as Huaxi and COBRE) are relatively high, they do not exceed the significance level (*p* < 0.05), which still shows the stable advantages of our method on various datasets. These results further verify the universality and effectiveness of our method on multiple datasets from a statistical perspective.

**Table 3:** The *t*-test *p*-value results of our method and the three best performing comparison methods (SPEA, PSO-MET and MTPSO) on ACC.

**Datasets**	**SPEA/our**	**PSO-MET/our**	**MTPSO/our**
COBRE	0.0015	0.0220	0.0490
Huaxi	0.0439	0.0133	0.0269
Nottingham	0.0037	0.0019	0.0249
Taiwan	0.0143	0.0195	0.0174
Xiangya	0.0016	0.0029	0.0428

### 4.6 Counterfactual explanations

**Figure 6:**
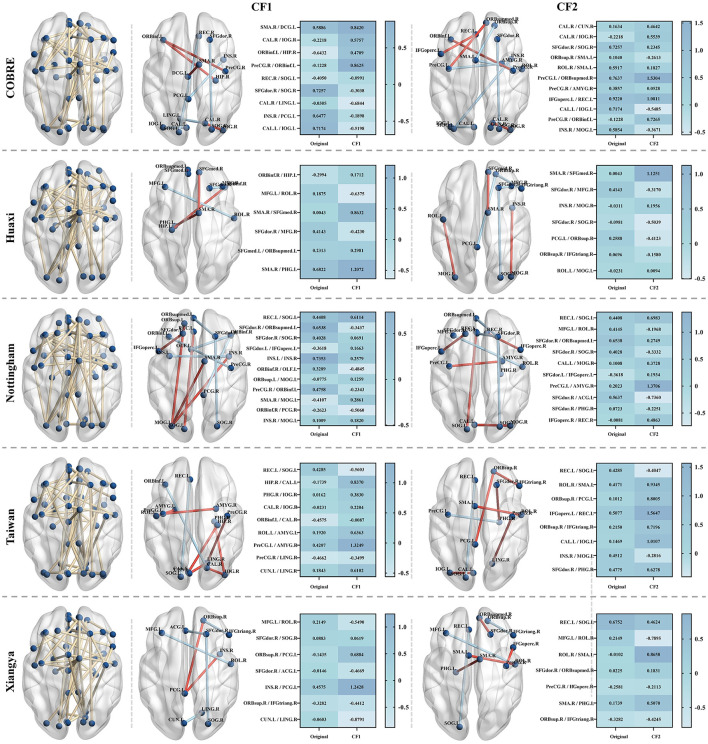
Counterfactual (CF) examples generated for randomly selected SZ patients in the test set in the five datasets. The yellow lines in the left brain map indicate the remaining functional connectivity (FC) after feature selection by our method, and the blue nodes indicate the corresponding brain regions. The brain maps in CF1 and CF2 show two counterfactual examples generated for the abnormal FC of the patients. The red lines indicate an increase in the FC value between the corresponding regions, and the blue lines indicate a decrease in the FC value. The heat map on the right shows the original FC value between the corresponding brain regions of the patients and the FC value after the counterfactual explanation.

In this set of experiments, we demonstrate how to generate a set of intuitive and diverse counterfactual (CF) examples for patients through the counterfactual explanation model. We provide counterfactual explanations by fine-tuning the abnormal FC value changes of patients, that is, adjusting the FC values between specific regions to make the patient's state closer to that of normal people. We generate two different counterfactual examples for SZ patients and present them in the form of brain maps and heat maps, as shown in Figure 6. It is not difficult to see that we can make the patient's state close to normal by only slightly adjusting the FC values between the corresponding regions. Specifically, in the Huaxi dataset, CF1 increases the FC values between ORBinf.R–HIP.L, SMA.R–SFGmed.R, SFGmed.L–ORBsupmed.L, and SMA.R–PHG.L from –0.2994, 0.0043, 0.2313, and 0.6822 to 0.1712, 0.8632, 0.2981, and 1.2072, and decreases the FC values between MFG.L–ROL.R and SFGdor.R–MFG.R from 0.1875 and 0.4143 to –0.6375 and –0.4230. In the Xiangya dataset, CF1 decreases the FC values between MFG.L–ROL.R, SFGdor.R–SOG.R, SFGdor.R–ACG.L, ORBsup.R–IFGtriang.R, and CUN.L–LING.R from 0.2149, 0.0883, –0.0146, –0.3282, and –0.0603 to –0.5490, 0.0619, –0.4669, –0.4412, and –0.8791, and increases the FC values between ORBsup.R–PCG.L and INS.R–PCG.L from –0.1435 and 0.4575 to 0.6884 and 1.2428, respectively. We find that the changes in functional connectivity (FC) after counterfactual interpretation remain stable within 1, without large-scale fluctuations, which further illustrates the robustness of our method. In addition, the role of FC changes in SZ patients has been observed in a large number of studies, such as Lynall et al. (2010), Fornito and Bullmore (2015), and Li et al. (2017).

## 5 Discussion

In this paper, we propose a multi-task feature selection method for SZ diagnosis, and combine it with the counterfactual explanation model to fine-tune the abnormal FC features of SZ patients to make their state closer to that of healthy individuals, thereby improving the accuracy of SZ classification and the interpretability of the model. To demonstrate the effectiveness of our method, we conduct empirical studies on five SZ datasets. Our results show that across the five datasets, 16 FC features are selected simultaneously. These shared FC features are mainly distributed in key brain regions such as the prefrontal cortex (PFC), cingulate gyrus (CC) and hippocampus (HIP), which are widely considered to be closely related to the pathological mechanism of SZ in previous studies. For example, the study by Minzenberg et al. (2009) shows that PFC dysfunction is closely related to executive function deficits in SZ patients. Whitfield-Gabrieli et al. (2009) find that SZ patients have significant abnormalities in FC in the default mode network (including CC), which is associated with cognitive dysfunction. Gangadin et al. (2021) and Li X.-W. et al. (2023) find that SZ patients have significant abnormalities in FC between HIP and other brain regions in the resting state. These results not only verify that the abnormal FC features screened out by our method under multiple datasets are consistent and stable, but also further confirm its potential value in the diagnosis and interpretation of SZ from a neurobiological perspective.

Although previous studies reveal a variety of brain FC abnormalities associated with SZ, there is still a lack of an interpretable diagnostic tool in the diagnosis of SZ. Our study proposes an innovative method that integrates multi-task feature selection and counterfactual explanation. To generate accurate counterfactual examples, we construct a counterfactual explanation model through three parts: loss function *loss*(·), distance function *dist*(·), and diversity index *diversity*(·). Specifically, *loss*(·) pushes counterfactual examples toward different predictions, *dist*(·) brings the counterfactual example closer to the original input, and *diversity*(·) increases the diversity of counterfactual explanations. We capture the brain regions where patients show abnormal FC features and slightly adjust the FC values between abnormal brain regions to make them closer to the normal state. This analysis method not only improves the interpretability of the classification model, but also provides an intuitive individual-level explanatory perspective for understanding brain FC abnormalities in SZ patients, which helps to identify potential intervention targets and promotes the application of precision medicine in the diagnosis of SZ.

However, the current study still has several limitations. First, we only use the AAL model to define brain regions. In the future, we use different templates to evaluate the effectiveness of our proposed method. Second, we have not yet established cooperation with clinical medical institutions and lack counterfactual change explanations reviewed by clinicians. We plan to introduce clinical validation to further demonstrate the practicality and effectiveness of the method. Finally, this study focuses on the SZ dataset and further verifies the generalization ability and application potential of the method on other brain disease datasets such as Alzheimer's disease and autism.

## 6 Conclusion

In this paper, we propose a robust feature selection method based on multi-task optimization for SZ identification, and explain the changes in brain functional connectivity caused by the disease through a counterfactual explanation model. Compared with traditional methods, our proposed method not only improves the recognition performance, but also provides an intuitive explanation for the prediction of SZ, and verifies the effectiveness of the method on five SZ datasets.

## Data Availability

The original contributions presented in the study are included in the article/supplementary material, further inquiries can be directed to the corresponding authors.
